# Optimization-based design of heat flux manipulation devices with emphasis on fabricability

**DOI:** 10.1038/s41598-017-06565-6

**Published:** 2017-07-24

**Authors:** Ignacio Peralta, Víctor D. Fachinotti

**Affiliations:** Centro de Investigación de Métodos Computacionales (CIMEC), Universidad Nacional del Litoral (UNL)/Consejo Nacional de Investigaciones Científicas y Técnicas (CONICET), Predio CCT-CONICET Santa Fe, Ruta Nac. 168, Paraje El Pozo, 3000 Santa Fe, Argentina

## Abstract

In this work, we present a new method for the design of heat flux manipulating devices, with emphasis on their fabricability. The design is obtained as solution of a nonlinear optimization problem where the objective function represents the given heat flux manipulation task, and the design variables define the material distribution in the device. In order to facilitate the fabrication of the device, the material at a given point is chosen from a set of predefined metamaterials. Each candidate material is assumed to be a laminate of materials with high conductivity contrast, so it is a metamaterial with a highly anisotropic effective conductivity. Following the discrete material optimization (DMO) approach, the fraction of each material at a given finite element of the mesh is defined as a function of continuous variables, which are ultimately the design variables. This DMO definition forces the fraction of each candidate to tend to either zero or one at the optimal solution. As an application example, we designed an easy-to-make device for heat flux concentration and cloaking.

## Introduction

The use of materials with unprecedented effective properties (the so-called “metamaterials”) to control the electromagnetic flux has led to major innovations in optics, electronics and communications^1, 2^. Taking advantages of the analogies between electromagnetism and thermodynamics^3^, very interesting, still academic, applications of metamaterials for heat flux manipulation were recently published, including inversion^4^, shielding^4, 5^, concentration^4, 6^, and cloaking^4, 7^. Of course, all these examples were effective to prove the potential of thermal metamaterials, but their practical application needs further research.

We identify two main obstacles to the practical use of thermal metamaterials. First, most of them^4–7^ were designed on the base of coordinate transformation, an approach inherited from electromagnetism^8^. Up to the authors’ knowledge, the transformation-based approach has been always applied to design metamaterials occupying geometrically simple domains, under homogeneous external heat flux, in order to accomplish a few specific tasks (shielding, concentration, inversion, cloaking). In other words, it has not been (cannot be?) applied to general problems.

To circumvent such limitations, we apply here an optimization-based methodology^9^, where the cost function defines an arbitrary heat flux manipulation task, to be accomplished in an arbitrary domain under arbitrary boundary conditions.

The other obstacle for real-life applications is the difficult fabrication of the devices, mainly because they need a precise metamaterial variation inside them. This was circumvented by Vemuri *et al*.^10^ by fabricating heat flux manipulating devices using a homogeneous laminate that was arranged in two different orientations at each fourth of the device. By this way, they approached as well as possible the thermal conductivity distribution required to accomplish the given task.

Agreeing with Vemuri *et al*., we consider the coarse discretization of the metamaterial spatial distribution to be the way to facilitate the fabrication of metamaterial devices. Then, we determine the geometry of the resulting homogeneous subdomains as solution of the problem of minimizing the error in the task accomplishment. If the material for each subdomain is chosen between only two metamaterials, this solution can be obtained by adapting techniques borrowed from topology optimization^11^. In case of a larger set of candidate materials, analogies can be found with multiphase topology optimization^12^. Particularly, we will define the fraction of each candidate material as a function of continuous design variables following the discrete material optimization (DMO) approach^13^, which allows to use efficient gradient-based optimization solvers.

However, the current problem has crucial differences with classical topology optimization problems. First, the objective function in topology optimization is either the material volume or the compliance^11^, linearly dependent on the design or the state variables, respectively. Meanwhile, the current objective represents the given task and is a highly nonlinear function of both the design and the state variables. Secondly, to consider the material volume (either as the objective function or as a constraint) is imperative for topology optimization but not in this case. Actually, it may be interesting to minimize or to limit the material volume (for instance, that of the most expensive material), but this is out of the scope of the current work.

Finally, we applied the current DMO-based approach for the design of an easy-to-make device for heat flux concentration and cloaking, as alternative to that one with continuously spatially variable metamaterial distribution we previously designed^9^.

## Heat flux Manipulation

### Steady thermal field as function of material distribution

Applying the finite element method (FEM), the temperature distribution in the domain Ω is approximated as

1 $$T({\bf{x}})={\bf{N}}({\bf{x}}){\bf{T}},\quad \forall {\bf{x}}\in {\rm{\Omega }},$$

where *N*_*i*_ is the shape function associated to node *i* and *T*_*i*_ is the temperature at this node. Assuming steady heat conduction, **T** is the solution of

2 $${\bf{K}}{\bf{T}}={\bf{F}},$$

where **K** and **F** are the conductivity matrix and the nodal heat flux vector, respectively, given by

3 $${\bf{K}}={\int }_{{\rm{\Omega }}}{{\bf{B}}}^{T}{\bf{k}}{\bf{B}}{\rm{d}}V,$$

4 $${\bf{F}}={\int }_{\partial {{\rm{\Omega }}}_{{\rm{q}}}}{q}_{{\rm{wall}}}{\bf{N}}{\rm{d}}S,$$

with ${B}_{ij}=\partial {N}_{j}/\partial {x}_{i}$, such that **BT** = grad*T*, **k** is the effective thermal conductivity tensor, and *q*_wall_ is the prescribed heat flux at the portion ∂Ω_q_ of the boundary of Ω.

**Figure 1:**
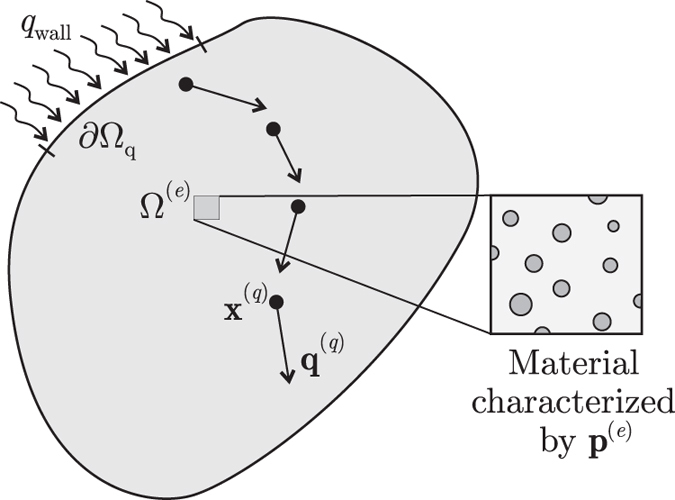
Heat flux manipulation problem in the domain **Ω** where the effective properties at each sub-domain **Ω** ^(*e*)^ depend on a set of parameters **p** ^(*e*)^.

Let **Ω** be a heterogeneous body, where the material is allowed to have an element-wise variation throughout the finite element mesh **Ω** = **Ω** ^(1)^∪**Ω** ^(2)^∪…∪**Ω** ^(*E*)^. Further, the effective material properties at the finite element **Ω** ^(*e*)^ are assumed to be determined by a finite number of parameters grouped in the vector ${{\bf{p}}}^{(e)}=[{p}_{1}^{(e)},{p}_{2}^{(e)},\ldots ,{p}_{M}^{(e)}]$, as shown in Fig. 1. So, the effective conductivity at **Ω** ^(*e*)^ is **k** ^(*e*)^ = **k**(**p** ^(*e*)^). This makes the conductivity matrix **K**, Equation (3), a function of the parameters of all the elements of the mesh, grouped in the vector ${\bf{P}}=[{p}_{1}^{\mathrm{(1)}},{p}_{2}^{\mathrm{(1)}},\ldots ,{p}_{M}^{(E)}]$. Consequently, the nodal temperature vector **T**, obtained as solution of Equation (2), is also a function of **P**, i.e. **T** = **T**(**P**).

### Task accomplishment as an optimization problem

At the point **x** ^(*q*)^ inside the finite element **Ω** ^(*q*)^, the heat flux is

$${{\bf{q}}}^{(q)}=-{\bf{k}}({{\bf{p}}}^{(q)}){\bf{B}}({{\bf{x}}}^{(q)}){\bf{T}}({\bf{P}})\equiv {{\bf{q}}}^{(q)}({\bf{P}}\mathrm{)}.$$

Now, to manipulate the heat flux inside Ω means to force the heat flux to take prescribed values ${\bar{{\bf{q}}}}^{(q)}$ at a series of points **x** ^(*q*)^ ∈ **Ω**, *q* = 1, 2,…, *Q*, see Fig. 1. To accomplish this task, we must find **P** such that

5 $${{\bf{q}}}^{(q)}({\bf{P}})={\bar{{\bf{q}}}}^{(q)},\quad \,{\rm{for}}\,{q}=1,2\ldots ,{Q}.$$

The search of **P** is constrained to an admissible design space ${\mathscr{D}}$. In general, it will not be possible to accomplish the given task for **P** ∈ ${\mathscr{D}}$. Then, let us do that as well as possible by solving the following nonlinear optimization problem:

6 $$\mathop{min}\limits_{{\bf{P}}\in D}{[\sum _{q=1}^{Q}{w}_{q}{\Vert {{\bf{q}}}^{(q)}({\bf{P}})-{\bar{{\bf{q}}}}^{(q)}\Vert }^{2}]}^{1/2},$$

where **P** plays the role of decision or design variables, and *w*_*q*_ is the weight given to the accomplishment of the task at the point **x** ^(*q*)^, with ${\sum }_{q}{w}_{q}=1$. If *w*_*q*_ = 1/*Q* = *constant*, the above objective function is the root mean square error (RMSE) in the accomplishment of the task.

In our previous work^9^, we designed a heat flux manipulating device allowing a continuous distribution of metamaterials. There, we recognized the need of further constraints to ensure the fabrication of the so-designed devices. These are the so-called manufacturing constraints in topology optimization^14^.

In this work, following Vemuri *et al*.^10^, we propose an alternative way of designing an easy-to-make heat flux manipulating device: it will be made of *M* distinct materials chosen from a predefined set; any material *m* = 1, 2, …, *M* may be a metamaterial itself, having a generally anisotropic conductivity **k**_*m*_.

### Parametrization of effective properties

The effective conductivity at the finite element Ω^(*e*)^ is defined by the mixture law:

7 $${{\bf{k}}}^{(e)}=\sum _{m=1}^{M}{f}_{m}^{(e)}{{\bf{k}}}_{m},$$

where ${f}_{m}^{(e)}$ is the fraction of material *m* at Ω^(*e*)^, such that $0\le {f}_{m}^{(e)}\le 1$ and ${\sum }_{i}{f}_{i}^{(e)}=1$. For easier fabricability, the material at Ω^(*e*)^ must be one of the candidates instead of a mixture of them, that is ${f}_{m}^{(e)}$ should be either one or zero. Assuming ${p}_{m}^{(e)}\equiv {f}_{m}^{(e)}$ as design variables, they are of integer type, so that the solution of the optimization problem (6) requires integer programming.

But integer programming is unaffordable in presence of a large number of design variables, as it is usually the case for fine enough finite element meshes. In this case, it is convenient to use a gradient-based optimization solver, which requires the design variables to be continuous. To this end, we make use of the “discrete material optimization” (DMO) technique proposed by Stegmann and Lund^13^, where ${f}_{m}^{(e)}$ is assumed to be a function of *M* continuous variables ${\rho }_{i}^{(e)}$, which play now the role of design variables, i.e. ${p}_{i}^{(e)}\equiv {\rho }_{i}^{(e)}$.

For optimal ${\rho }_{i}^{(e)}$, ${f}_{m}^{(e)}$ must tend to one or zero. Stegmann and Lund^13^ studied various choices for the function ${f}_{m}^{(e)}$, comparing them in terms not only of their closeness to one or zero at the optimal solution but also of their effect on the convergence to this solution, recommending the definition to be used in this work:

8 $$\begin{array}{rcl}{f}_{m}^{(e)} & = & {f}_{m}({\rho }_{1}^{(e)},\ldots ,{\rho }_{M}^{(e)})\\  & = & \frac{{f}_{m}^{\ast }({\rho }_{1}^{(e)},\ldots ,{\rho }_{M}^{(e)})}{\sum _{i=1}^{M}{f}_{i}^{\ast }\,({\rho }_{1}^{(e)},\ldots ,{\rho }_{M}^{(e)})},\quad \,{\rm{with}}\,{f}_{i}^{\ast }({\rho }_{1}^{(e)},\ldots ,{\rho }_{M}^{(e)})\\  & = & {({\rho }_{i}^{(e)})}^{p}\prod _{j=\mathrm{1,}j\ne i}^{N}[1-{({\rho }_{j}^{(e)})}^{p}].\end{array}$$

The above equation defines the fraction of material *m* at the finite element *e* as a function of the continuous design variables ${\rho }_{1}^{(e)},\ldots ,{\rho }_{M}^{(e)}$. Since this definition automatically yields ${\sum }_{i}{f}_{i}^{(e)}=1$, these are not longer constraints for the continuous optimization problem^6^, which is now subject to bound constraints only: $0\le {\rho }_{i}^{(e)}\le 1$. Like in topology optimization^11^, intermediate values of ${f}_{m}^{(e)}$ are penalized by setting *p* ≥ 3 in Equation (8).

### Choice of candidate materials

Metamaterials made of alternating sheets (i.e. laminates) of materials with markedly distinct conductivity exhibit a highly anisotropic effective conductivity, making them a popular choice for heat flux manipulation^4–6, 10^.

Following Vemuri *et al*.^10^, in the examples to be developed in this work we will use a laminate of alternating, equally-thick sheets of copper and polydimethylsiloxane (PDMS), with isotropic conductivities *k*_copper_ = 398 Wm^−1^K^−1^ and *k*_PDMS_ = 0.27 Wm^−1^K^−1^. Assuming such laminate as an effective thermal medium, its principal conductivities are

9 $${k}_{\lambda \lambda }=\frac{{k}_{{\rm{copper}}}+{k}_{{\rm{PDMS}}}}{2}=199.13\,{{\rm{Wm}}}^{-1}{{\rm{K}}}^{-1},\quad \quad {k}_{\tau \tau }=\frac{2{k}_{{\rm{copper}}}{k}_{{\rm{PDMS}}}}{{k}_{{\rm{copper}}}+{k}_{{\rm{PDMS}}}}=0.54\,{{\rm{Wm}}}^{-1}{{\rm{K}}}^{-1},$$

where *λ* and *τ* are the principal axes in-plane and normal to the sheets, respectively. Above equation is valid if the layers have small thickness and high conductivity contrast^15^. Here, the conductivity contrast between layers is very high (*k*_copper_/*k*_PDMS_ = 1474). Concerning the layer thickness, being the current macroscale dimensions on the order of centimeters, it should be on the order of 1 mm.

Finally, as candidate materials to build a device for heat flux manipulation, we adopt this copper-PDMS laminate with different orientations *θ* (angle between *λ* and *x* axes).

## Validation

**Figure 2:**
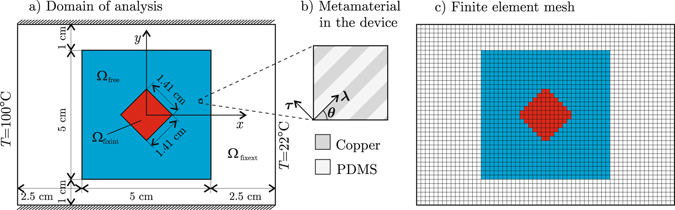
Validation example: (**a**) domain of analysis, (**b**) metamaterial used to build the device, (**c**) finite element mesh of **Ω**; the blue elements belong to the device, and the red ones have heat flux prescribed to be null.

For the purpose of validating the proposed DMO-based methodology for the design of heat flux manipulating devices, let us reproduce the simple device for heat flux shielding proposed by Vemuri *et al*.^10^. Given a plate **Ω** originally filled with 40% nickel steel (with isotropic conductivity *k*_ns_ = 10 Wm^−1^K^−1^), let us design a device occupying the region **Ω**_free_ to block the heat flux in the central region **Ω**_fixint_ (see Fig. 2). Further, let us do it as well as possible by using only two candidate materials: material 1 is the copper-PDMS laminate with *θ* = 45°, and material 2 is the same laminate with *θ* = 135°.

The domain **Ω** is discretized using 60 × 42 = 2520 bilinear square finite elements, as shown in Fig. 2c, including 840 elements inside the device **Ω**_free_ (the blue ones in that figure).

### Accomplishment of the shielding task

The shielding task is prescribed by setting ${\bar{{\bf{q}}}}^{(q)}={\bf{0}}$ at the 60 elements in **Ω**_fixint_ (the red ones in Fig. 2c). Then, the minimization problem^6^ takes the form:

10 $$\mathop{min}\limits_{{\bf{P}}}{[\frac{1}{60}\sum _{q|{{\rm{\Omega }}}^{(q)}\in {{\rm{\Omega }}}_{{\rm{fixint}}}}{\Vert {{\bf{q}}}^{(q)}({\bf{P}})\Vert }^{2}]}^{\mathrm{1/2}}.$$

This is a non-linear optimization problem with 1680 continuous design variables grouped in the vector ${\bf{P}}=[{\rho }_{1}^{\mathrm{(1)}},{\rho }_{2}^{\mathrm{(1)}},\ldots ,{\rho }_{2}^{\mathrm{(840)}}]$, subject to the bound constraints 0 ≤ *P*_*i*_ ≤ 1.

### Results

The non-linear constrained optimization problem (10) is solved using the IPOPT interior point algorithm^16^. Further, we used a density-filtering technique^17^ to avoid checker-board type instabilities.

**Figure 3:**
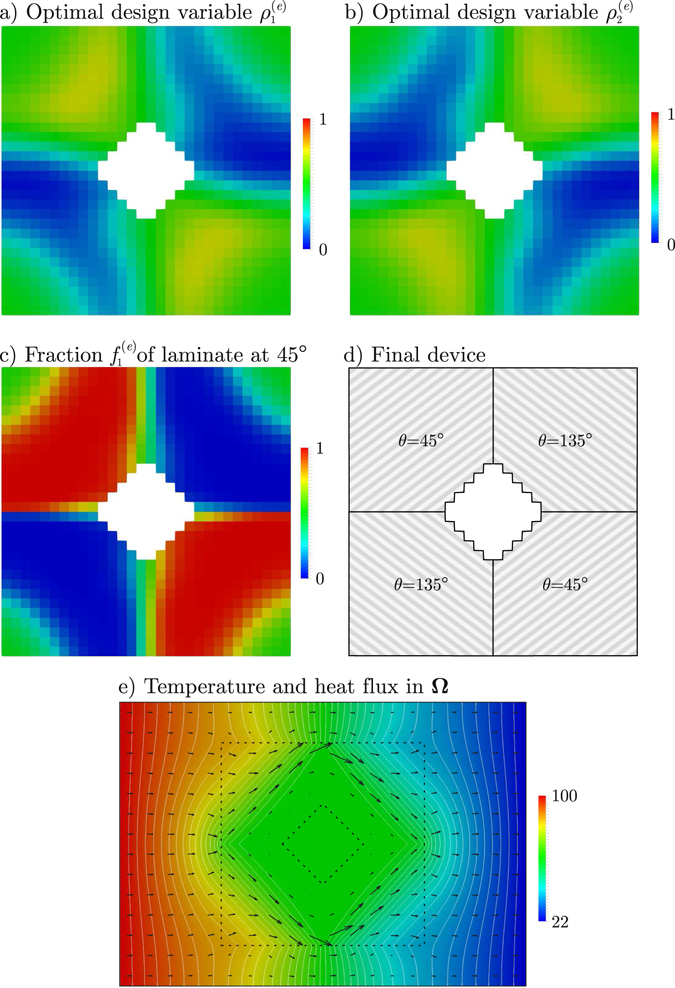
Validation example: (**a**) optimal design variable ${\rho }_{1}^{(e)}$, (**b**) optimal design variable ${\rho }_{2}^{(e)}$, (**c**) fraction of laminate at 45° for optimal ${\rho }_{1}^{(e)}$ and ${\rho }_{2}^{(e)}$, (**d**) actual device, (**e**) temperature (colormap with isolines every 2 °C) and heat flux (arrows) in the whole plate.

Figure 3a and b show the optimal design variables ${\rho }_{1}^{(e)}$ and ${\rho }_{2}^{(e)}$ inside the device. Then, using Equation (8) we compute the corresponding fraction ${f}_{1}^{(e)}$ of copper-PDMS laminate at 45° (i.e. material 1), shown in Fig. 3c; the fraction at 135° is simply ${f}_{2}^{(e)}=1-{f}_{1}^{(e)}$. The device having such material distribution accomplishes the shielding task with high accuracy: RMSE = 1.536 × 10^−4^||**q**_0_||, being ||**q**_0_|| = 7.8 kWm^−2^ the magnitude of the heat flux in the plate without the device.

The material distribution in Fig. 3c is mostly free of “grey zones” (those where the material is neither of the candidates but a mixture of them). To completely avoid “grey zones”, recourse can be made to “black-and-white” filters^18^. In this work, a simple *a posteriori* “black-and-white” filter is applied: the material at **Ω** ^(*e*)^ is that having the greatest fraction. The resultant piecewise homogeneous metamaterial distribution in the device is shown in Fig. 3d, using which the temperature and heat flux distribution in the whole domain **Ω** are those depicted in Fig. 3e. The shielding task is still very well accomplished: RMSE = 9.454 × 10^−3^||**q**_0_||.

Figure 3d is actually the DMO-designed shielding device, where each quadrant is made of the copper-PDMS laminate with either divergent or convergent orientation upstream and downstream the shielded domain **Ω**_fixint_. This design coincides with Vemuri *et al*.’s^10^, and gives unsurprisingly the best way of forcing the heat flux off **Ω**_fixint_ using the two given materials.

## Application to a heat concentration and cloaking problem

**Figure 4:**
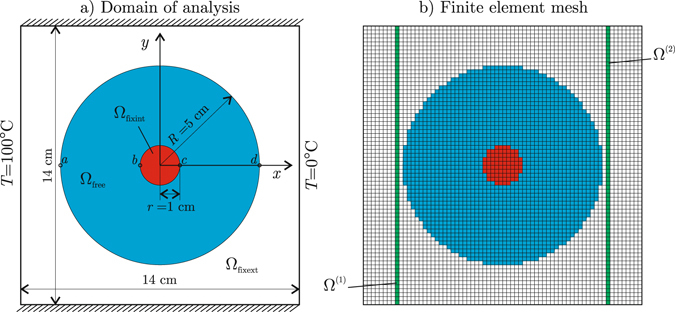
Heat flux concentration and cloaking example: (**a**) domain of analysis, (**b**) finite element mesh of **Ω**; the blue elements belong to the device, while the red and the green ones have prescribed heat flux for concentration and cloaking, respectively.

Now, let us apply the current methodology for the design of a device for heat concentration and cloaking as alternative to that one we designed using an optimization-based continuous metamaterial distribution^9^ as well as to that one designed by Chen and Lei^6^ using the transformation-based approach. Figure 4a shows the domain of analysis **Ω**, originally filled with 40% -nickel steel, with isotropic thermal conductivity *k*_ns_ = 10 Wm^−1^K^−1^. At this point, the plate undergoes a homogeneous heat flux **q**_0_ with magnitude ||**q**_0_|| = 7.14 kWm^−2^ and direction +*x*.

Then, let us design a device occupying the region **Ω**_free_ to accomplish the following simultaneous tasks: 1) to concentrate the heat in **Ω**_fixint_, and 2) to keep the heat flux unaltered in **Ω**_fixext_. Three candidate materials will be used, all of them consisting of the current copper-PDMS laminate but differently oriented: material 1 with *θ* = 45°, material 2 with *θ* = 135°, and material 3 with *θ* = 0°.

The domain **Ω** is discretized using 70 × 70 = 4900 bilinear square finite elements, as shown in Fig. 4b, including 1896 elements inside the device **Ω**_free_ (the blue ones in this figure).

### Accomplishment of the concentration and cloaking tasks

The cloaking task ${\bar{{\bf{q}}}}^{(q)}={{\bf{q}}}_{0}$ is prescribed at the 140 elements in the stripes Ω^(1)^ ⊂ Ω_fixext_ and Ω^(2)^ ⊂ Ω_fixext_ (the green ones in Fig. 4b), while the heat concentration task is forced by setting ${\bar{{\bf{q}}}}^{(q)}={{\bf{q}}}_{{\rm{conc}}}$ for the 80 elements in **Ω**_fixint_ (the red ones in Fig. 4b); here, we adopted **q**_conc_ = (*R*/*r*)**q**_0_, being *R*/*r* = 5 the ratio between the outer and the inner radii in the device. Then, the minimization problem (6) takes the form:

11 $$\mathop{{\rm{\min }}}\limits_{{\bf{P}}}[\frac{1}{220}(\sum _{q|{{\rm{\Omega }}}^{(q)}\in {{\rm{\Omega }}}^{\mathrm{(1)}}\cup {{\rm{\Omega }}}^{\mathrm{(2)}}}{\Vert {{\bf{q}}}^{(q)}({\bf{P}})-{{\bf{q}}}_{0}\Vert }^{2}+{\sum _{q|{{\rm{\Omega }}}^{(q)}\in {{\rm{\Omega }}}_{{\rm{fixint}}}}{\Vert {{\bf{q}}}^{(q)}({\bf{P}})-{{\bf{q}}}_{{\rm{conc}}}\Vert }^{2})]}^{\mathrm{1/2}}.$$

**Figure 5:**
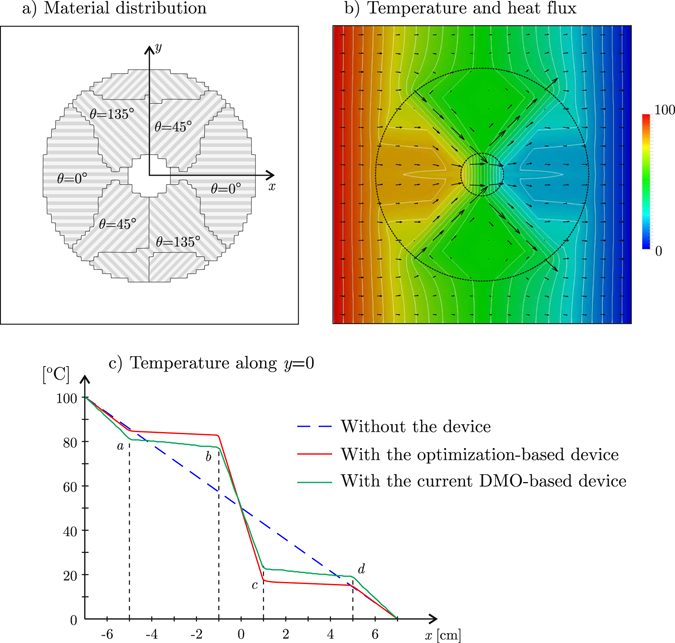
Heat flux concentration and cloaking example: (**a**) DMO-designed device, (**b**) temperature (colormap with isolines every 4 °C) and heat flux (arrows) in the whole plate, (**c**) temperature along *y* = 0 for the current DMO-designed device and the optimization-designed one with continuous metamaterial distribution^9^.

This is a non-linear optimization problem with 5688 continuous design variables grouped in the vector ${\bf{P}}=[{\rho }_{1}^{\mathrm{(1)}},{\rho }_{2}^{\mathrm{(1)}},{\rho }_{3}^{\mathrm{(1)}},\ldots ,{\rho }_{3}^{\mathrm{(1896)}}]$, subject to the bound constraints 0 ≤ *P*_*i*_ ≤ 1. Using IPOPT^16^ as optimization solver, together with density filtering^17^ to avoid checkerboard instabilities, we compute first the optimal distribution of the design variables ${\rho }_{1}^{(e)}$, ${\rho }_{2}^{(e)}$ and ${\rho }_{3}^{(e)}$, which are then used to compute the material fractions ${f}_{1}^{(e)}$, ${f}_{2}^{(e)}$ and ${f}_{3}^{(e)}$ according to Equation (8). Finally, assuming that the material at an element is that having the highest fraction, we obtain the device depicted in Fig. 5a, using which the temperature and heat flux in the whole plate are those shown in Fig. 5b. As it can be observed in Fig. 5a, by properly orienting the laminate, the heat flux is guided as directly as possible to **Ω**_fixint_. Quantitatively, the RMSE for concentration is 0.284||**q**_conc_||. Regarding cloaking, the RMSE is similar: 0.277||**q**_0_||.

The current easy-to-make DMO-designed device given by Fig. 5a performs almost as well as the hard-to-make device we designed using a continuous metamaterial distribution^9^ (where the thickness of the alternating copper and PDMS layers and their orientation vary throughout the device), specially for concentration. This is clearly apparent from Fig. 5c, showing the temperature distribution along the line *y* = 0 for both devices.

Now, after Chen and Lei^6^, let us consider a concentration efficiency index defined as *f* = (*T*_*c*_ − *T*_*b*_)/(*T*_*d*_ − *T*_*a*_), where *b* and *c* are points located in the boundary of the heat concentration region **Ω**_fixint_, and *a* and *d* are points located at the outer boundary of the device **Ω**_free_ (see Fig. 4a). For the current device, we obtain *f* = 88.37%. This is slightly better than the efficiency of the device fabricated by Chen and Lei^6^, made of 100 radial copper-PDMS laminates.

Note that the deal between both tasks can be changed by tuning the weights *w*_*q*_, which were assumed to be constant in this example. Actually, cloaking is not usually the main task but most of the devices found in the literature^4, 6^ perform it as a collateral effect of their transformation-based design.

## Conclusions

Having already demonstrated the potentiality of the optimization-based approach for the design of metamaterial devices for heat flux manipulation^9^, we faced in this work a crucial deterrent for real-life applications of such devices: their fabricability. To this end, easy-to-make heat flux manipulating devices are designed as assemblages of homogeneous subregions made of materials chosen from a predefined set of candidates. Each candidate may be a metamaterial itself, having a highly anisotropic thermal conductivity.

The optimal geometry of these subregions is determined as solution of a non-linear optimization problem where the desired heat flux manipulating task is the objective function. Then, we used the Discrete Material Optimization (DMO) approach to define the material fraction as a function of continuous design variables that rapidly tend to 0 or 1 in order to ensure that the material at a point is one of the candidates and not a mixture of them.

Making so easy to manipulate the heat flux, we hope this work shows the way for the expansion of the use of thermal metamaterials in industry.
